# Editorial: Optimal omegas

**DOI:** 10.3389/fnut.2024.1529722

**Published:** 2024-12-31

**Authors:** Emma Derbyshire, K. E. Lane, Ivana Djuricic

**Affiliations:** ^1^Nutritional Insight Limited, London, United Kingdom; ^2^Liverpool John Moores University, Liverpool, United Kingdom; ^3^University of Belgrade, Belgrade, Serbia

**Keywords:** health, omega, eicosapentaenoic acid, docosahexaenoic acid, novel foods

The Frontiers “*Optimal omegas*” Research Topic was assembled to provide a scientific update on field human health studies, the modulation of metabolic pathways, traditional and novel dietary sources of omega-3 fatty acids, sustainable production of foods providing omega-3 fatty acids and bioavailability studies.

Trends toward sustainable plant-based diets mean that modern dietary staples are shifting with momentum, leading to ramifications for intakes of omega-3 fatty acids and the essential fatty acid metabolic pathway. Subsequently, there are growing demands for sustainable food-derived sources of omega-3 (ω-3) fatty acids.

Two publications in the Research Topic used National Health and Nutrition Examination Survey (NHANES) data. Li Y. et al. identified that omega-3 and omega-6 polyunsaturated fatty acids were negatively associated with neutrophil-lymphocyte ratio, white blood cell counts, systemic immune-inflammation index and platelet-lymphocyte ratio. Interestingly, Yan et al. found that omega-3 intakes exceeding 2.05 g and ω-6 intakes ranging from more than 11.42 g to ≤ to 19.16 g lowered frailty risk amongst middle-aged and elderly individuals.

An updated systematic review of randomized controlled trials found that eicosapentaenoic acid (EPA) and docosahexaenoic acid (DHA) both lower triglyceride levels, with docosahexaenoic acid having a greater effect (Choi and Calder). Li K. et al. found that mussel oil was more potent than fish oil in preventing atherosclerosis, with the downregulation of p38MAPK/NF-κB signaling pathway being one plausible mechanism. Derbyshire et al. discussed potential ways forward focusing on innovative delivery methods to utilize omega-3 long-chain polyunsaturated fatty acid rich oils including the use of fortification strategies, bioengineered plants, microencapsulation, and microalgae.

A pilot study in patients with the homozygous ancestral (minor) FADS1 genotype demonstrated that FADS1 genotypes significantly decreased blood levels of ω-6 polyunsaturated fatty acids (PUFAs), particularly arachidonic acid (AA) without having a notable impact on the ω-3 PUFAs such as EPA and DHA, liver fat content and AA-derived lipid mediators (Rabehl et al.).

Macura et al. found the resilience of DHA homeostasis in the retina and retinal pigmented epithelium (RPE) of 2-month-old mouse offspring, even following high-dose fish oil supplementation during pregnancy and lactation. Another significant finding in this study was an upregulation of major facilitator superfamily domain-containing protein (Mfsd2a), a key DHA transporter and transcytosis regulator during development. The post-market cohort study showed that AlmegaPL^®^, an EPA-only polar lipid supplement derived from the microalga Nannochloropsis, effectively reduced triglycerides (TG) and remnant cholesterol (RC) in a real-world consumer setting (Ganuza et al.).

A clinical trial conducted by Rafieipoor et al. revealed that ω-3 supplementation (3 g/day for 2 months) did not have a significant effect on managing chronic kidney disease-associated pruritus (CKD-aP) in patients undergoing hemodialysis.

Overall, these articles have provided some valuable field insights within the omega scientific field. In particular, non-animal derived sources of omegas is an exciting area to monitor for the future and one for which ongoing research is highly valuable.

